# Effect of Swimming on Clinical Functional Parameters and Serum Biomarkers in Healthy and Osteoarthritic Dogs

**DOI:** 10.1155/2014/459809

**Published:** 2014-01-09

**Authors:** Korakot Nganvongpanit, Sikhrin Tanvisut, Terdsak Yano, Prachya Kongtawelert

**Affiliations:** ^1^Animals Bone and Joint Research Laboratory, Department of Veterinary Biosciences and Public Health, Faculty of Veterinary Medicine, Chiang Mai University, Chiang Mai 50100, Thailand; ^2^Department of Food Animals, Faculty of Veterinary Medicine, Chiang Mai University, Chiang Mai 50100, Thailand; ^3^Thailand Excellence Center for Tissue Engineering, Department of Biochemistry, Faculty of Medicine, Chiang Mai University, Chiang Mai 50000, Thailand

## Abstract

This study aimed to determine whether swimming could improve function of osteoarthritic joints in canine hip OA. Fifty-five dogs were categorized into three groups. The OA with swimming group (OA-SW; *n* = 22), the healthy (non-OA; *n* = 18) with swimming group (H-SW), and the healthy (non-OA; *n* = 15) without swimming group (H-NSW). All animals were allowed to swim for a total of 8 weeks (2-day period, 3 cycles of swimming for 20 minutes, and resting period for 5 minutes in each cycle). Three ml of blood was collected every 2 weeks for evaluation of the levels of biomarkers for OA, including chondroitin sulfate epitope WF6 (CS-WF6) and hyaluronan (HA). Clinical evaluation of the OA-SW group found that most parameters showed improvement (*P* < 0.01) at week 8 compared to pretreatment, while pain on palpation was improved (*P* < 0.01) at week 6. The relative level of serum CS-WF6 in the OA-SW group was found to be significantly different (*P* < 0.01) at weeks 6 and 8 compared with the preexercise. The levels of serum HA of the H-SW group in weeks 2–8 were significantly (*P* < 0.01) higher than preexercise. Conclusion, swimming over 2-day period, 8 weeks continually, can improve the function of OA joint.

## 1. Introduction

One of the most prevalent musculoskeletal disorders in canines is osteoarthritis (OA). Dogs with OA show clinical signs including lameness, increasing immobility, and muscle weakness, which can lead to a reduction in quality of life. Although OA cannot be cured, long-term management of the disease can be very rewarding for the veterinarian and pet owner. Managing pain with pain medications is an essential first step, including nonsteroidal anti-inflammatory drugs and chondroprotective drugs [1]. There are also physical modalities available for pain reduction [2]. Weight management and nutritional joint support are also important aspects of managing OA. Moreover, physical rehabilitation is a great way to improve mobility and keep dogs active as they age [3]. And finally there is surgical management, for example, chondrocyte transplantation, arthrodesis, and arthroplasty [4, 5].

Rehabilitation protocols in the veterinary field are modeled after those proven to be beneficial in people. Although much research has been published on the use of swimming as physical therapy for humans, there have been few controlled studies on swimming as a treatment protocol for dogs. However, many reports have shown the advantages of rehabilitation programs for dogs [6–12]. Current guidelines recommend rehabilitation methods as a first-line option for OA management. Reflect the fact that swimming, walking, and massage are not the only modalities employed in rehabilitation, particularly for OA. Aquatic exercise is suitable for OA patients—dogs and humans as well. The buoyancy, hydrostatic pressure, viscosity, resistance, and surface tension of water increase the efficacy of the exercise [13]. These properties of water have a positive effect, resulting in increased muscle mass, strength, and endurance, as well as decreased pain during movement [14, 15]. Water buoyancy significantly decreases contact force and stress on weight-bearing joints, bones, and muscles, which in turn reduces pain [16].

Due to the lack of effective monitoring methods of joint homeostasis during swimming in OA dogs, this study aimed to determine if swimming could improve the function of OA in canine hip joints. Moreover, two serum biomarkers, chondroitin sulfate epitope WF6 (CS-WF6) and hyaluronan (HA), were used to monitor joint homeostasis during the 8-week swimming program. A monoclonal antibody CS-WF6, which recognizes a native epitope in CS chain, and serum HA have been studied as a biomarkers of disease progression, since significantly increased levels were reported in cases of osteoarthritis [4]. For this our hypothesis is based on that if swimming could improve function of OA joint, clinical signs and biomarker level should improve.

## 2. Materials and Methods

### 2.1. Animals

The experimental protocol was approved by the Faculty of Veterinary Medicine and the Ethics Committee, Chiang Mai University, Thailand. Fifty-five dogs with a body condition score [17, 18] between 3–6 out of 9, included German shepherd (*n* = 3), Golden Retriever (*n* = 12), Labrador Retriever (*n* = 18), Beagle (*n* = 2), Pug (*n* = 5), Shih Tzu (*n* = 4), French Bulldog (*n* = 2), American Pit Bull Terrier (*n* = 5), and Bangkaew (*n* = 4). The dog were randomized and categorized into three groups. Twenty-two dogs were in the OA with swimming group (OA-SW), consisting of 9 males and 13 females, 47.62 ± 23.21 months old and 25.52 ± 10.82 kg. The healthy with swimming group (H-SW) had 18 non-OA dogs consisting of 8 males and 10 females, 48.33 ± 21.26 months old and 26.00 ± 9.14 kg. The third group, the healthy without swimming group (H-NSW) had 15 non-OA dogs consisting of 8 males and 7 females, 38.69 ± 20.73 months old and 19.85 ± 13.20 kg. The healthy 33 dogs were categorized into swimming group (H-SW) and without swimming group (H-NSW) using computer program.

### 2.2. Inclusion/Exclusion Criteria for Canine Osteoarthritis

Dogs with clinical signs of chronic lameness (more than 1 month), stiffness and joint pain, and radiological evidence of OA of the hip were eligible. Dogs were examinated by orthopedic veterinarian to confirm OA, previously entrance to this study. All OA dogs were categorized into grades 1–3 according to Table 1. Animals which were grade-4 OA dogs, pregnant, and receiving medication or which had hepatic, cardiovascular, gastrointestinal, or neurological disease were excluded. Dogs with lameness due to lumbosacral instability, infection, immune disease, or fractures and dogs which had previously received drug or dietary supplements for OA treatment were also excluded. Moreover, animals were not allowed to have received nonsteroidal anti-inflammatory drugs (NSAIDs) or chondroprotective drugs for 1 month preexperiment, as well as during the experimental period.

### 2.3. Swimming Protocol

An outdoor pool was used for aquatic exercise, with a water temperature between 30–35°C. All dogs were allowed to swim for a total of 8 weeks in order to collect the data. Swimming times were measured each week over a 2 d period. The daily protocol consisted of three cycles of swimming for 20 min followed by a 5 min resting period [19].

### 2.4. Assessment Protocol

Clinical signs, range of motion, and blood collection were performed before starting exercise program and repeated every 2 weeks until week 8. Two veterinarians recorded the severity of clinical signs and range of motion (ROM) using goniometer every 2 weeks using an ordinal scoring system (Table 2) [20, 21]; all veterinarians were blinded to animal. Radiographs of the hip joints were taken prior to the study and at the end of the study period at week 8 and were interpreted by the two veterinarians using the scoring system described in Table 1 [21, 22] which blinded as well. Three mL of blood was collected from each dog's cephalic vein every 2 weeks for evaluation of the level of biomarkers for OA [4, 21, 23].

### 2.5. Clinical Score

Efficacy of the treatment was assessed by means of a clinical scoring system [20, 21] which assessed a specific animal's lameness, joint mobility, pain on palpation, weight-bearing, and overall score of clinical condition. The dogs walked and trotted 12 meters (6 meters for evaluate), 3 times each, for evaluation of lameness by two veterinarians. This was followed by palpation of the hip joint for joint mobility and pain evaluation; the palpation was performed by two veterinarians 30 min apart.

### 2.6. Radiographs

Radiographs were taken for each animal, at enrollment and after 8 weeks of treatment, by the same technician using a standard X-ray machine. Ventrodorsal radiographs were obtained with the dog's hip and leg in the full extension position. Repositioning of the dog for subsequent radiography was guided by the original film, and the same radiographic settings (i.e. kV, mA and ms) were used. All radiographs in a set (2 films) for each dog were evaluated concurrently by two veterinarians using the criteria in Table 1. Only dogs with hip joint OA of grades 1–3 were used as subjects of this study.

### 2.7. Blood Collection

Three mL blood samples were taken in the morning before feeding the dogs. One mL blood samples from each dog were kept in anticoagulant (100 IU/mL heparin) for a complete blood count (CBC). Two mL blood samples were centrifuged at 10,000 ×g for 15 min to obtain the serum; this was kept frozen at −20°C until blood chemical tests and biomarker assay were performed.

### 2.8. Hematology and Biochemistry

CBCs and blood chemistry tests were conducted at the Small Animal Hospital, Faculty of Veterinary Medicine, Chiang Mai University, Chiang Mai, Thailand. The blood samples were analyzed for CBC, including hematocrit and hemoglobin levels, red blood cell count, white blood cell count (WBC), and platelet count. Two mL of serum was analyzed for blood chemicals, including aspartate aminotransferase (AST), alanine aminotransferase (ALT), blood urea nitrogen (BUN), and creatinine.

### 2.9. Biomarker Assay

ELISA (enzyme-linked immunosorbent assay) was used as a biomarker assay, following previous studies performed by our research group [4, 21, 23, 24] at Thailand Excellence Center for Tissue Engineering, Department of Biochemistry, Faculty of Medicine, Chiang Mai University, Chiang Mai, Thailand.

#### 2.9.1. ELISA-Based Assay for the Chondroitin Sulfate WF6 Epitope

A quantitative two-step ELISA was developed based on the results from an initial study that characterised the epitopes recognized by the monoclonal antibody WF6. Diluted canine serum samples, 1 : 5 in 6% BSA-TE (bovine serum albumin-tris/EDTA) buffer, were added to 1.5 mL plastic tubes containing an equal volume of monoclonal antibody WF6 (cell culture supernatant, 1 : 200 dilution in TE buffer). The standard used was embryonic shark skeletal cartilage aggrecan (the A1D1 fraction) at different concentrations (19–10,000 ng/mL) in 6% BSA-TE buffer. After incubation at 37°C for 1 h, the samples (or standard) mixed with WF6 were added to a microtiter plate previously coated with shark skeletal aggrecan (the A1 fraction) (100 *μ*L/well at 10 *μ*g/mL); the samples were blocked with 1% BSA. The plates were incubated at 37°C for 1 h, and the wells were then washed with TE buffer. Peroxidase-conjugated anti-mouse IgM antibody (Sigma-Aldrich, St. Louis MO, USA) was then added (100 *μ*L/well; 1 : 2,000 dilution in TE buffer). After incubation at 37°C for a further 1 h, the amount of bound peroxidase was determined using OPD (*o*-phenylenediamine dihydrochloride) substrate (Sigma-Aldrich). The plates were read at 492–690 nm. The WF6 epitope concentration in the samples was calculated from the standard curve.

#### 2.9.2. ELISA-Based Assay for Hyaluronan

An ELISA assay was developed for determining hyaluronan (HA) in serum, based on previous work with HA-binding proteins. Canine serum samples or standard HA (Healon) at various concentrations (19–10,000 ng/mL in 6% BSA-PBS, pH 7.4) were mixed with an equal volume of biotinylated HABPs (hyaluronan binding proteins) derived from bovine articular cartilage (1 : 200 in 0.05 M Tris-HCl buffer, pH 8.6). After incubation at room temperature for 1 h, the samples (100 *μ*L) were added to microplate wells previously coated with human umbilical cord HA (Sigma-Aldrich) (100 *μ*L/well at 10 *μ*g/mL); they were then blocked with 1% BSA (150 *μ*L/well). After further incubation at room temperature for 1 h, the wells were washed with PBS-Tween buffer. Peroxidase-conjugated anti-biotin antibody (Zymed, South San Francisco CA, USA) (1 : 2,000 dilution, 100 *μ*L/well in PBS) was added next. The plate was incubated at room temperature for a further 1 h, and the bound peroxidase was determined using OPD substrate. The plates were read at 492–690 nm. The amount of HA in the samples was calculated from the standard curve.

### 2.10. Statistical Analysis

The radiographic and clinical sign scores were calculated as mean ± SD. Nonparametric 2-sample Mann-Whitney procedure was used to test for differences before and after treatment. The results of serum CS-WF6 and HA analysis are presented as mean of relative change. Nonparametric 2-sample Mann-Whitney procedure was also used to test for differences between weeks 0, 2, 4, 6, and 8. Relative data were analyzed using the SAS version 8.0 software package; *P* ≤ 0.01 was considered to be significant.

## 3. Results

All dogs enrolled in the trial had hemogram and biochemical profile results within the reference range throughout the trial (data not shown). Twenty-two out of 77 dogs withdrew from the study due to various reasons: 10 dogs left because of illness, 5 dogs moved to another province, 2 dogs died from car accidents, and 4 dogs were unable to swim with sufficient frequency. Ultimately, 55 dogs served as subjects in this study.

Clinical evaluation of the OA-SW group found that nearly all parameters (lameness, joint mobility, weight bearing, and overall score) showed significant improvement (*P* < 0.05) at week 8 compared to pretreatment, while pain on palpation was significantly improved (*P* < 0.05) at week 6 (Table 3). For range of motion (ROM) evaluation, both extension and flexion of the hip joint were found to be significantly improved (*P* < 0.05) in the OA-SW and H-SW groups at week 8 compared to pretreatment, while the control group (H-NSW) showed no difference (Table 4).

**Figure 1:**
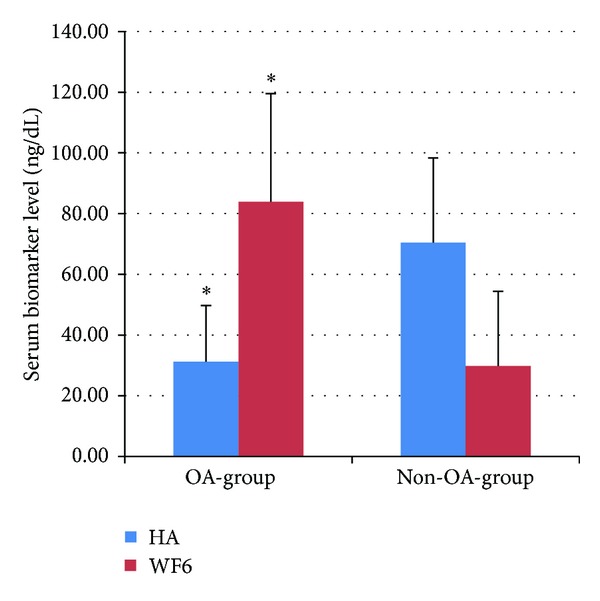
Mean (±SD) serum levels of biomarkers hyaluronan (HA) and chondroitin sulfate epitope (CS-WF6). *indicates a significant difference for the same biomarker between groups (*P* < 0.05).

**Figure 2:**
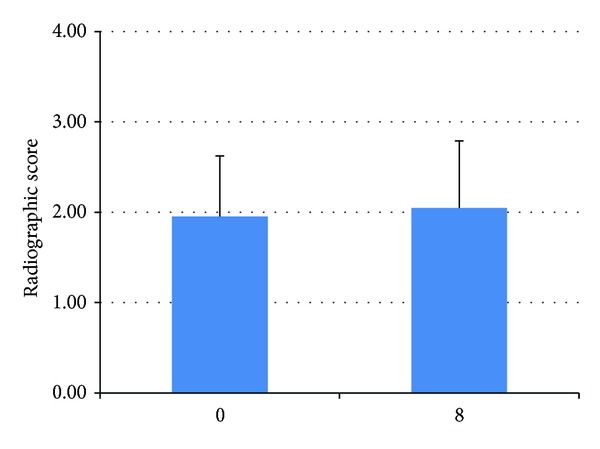
Mean (±SD) scores of radiographic images. The values were not significantly different between 0 and 8 weeks (*P* > 0.05).

**Figure 3:**
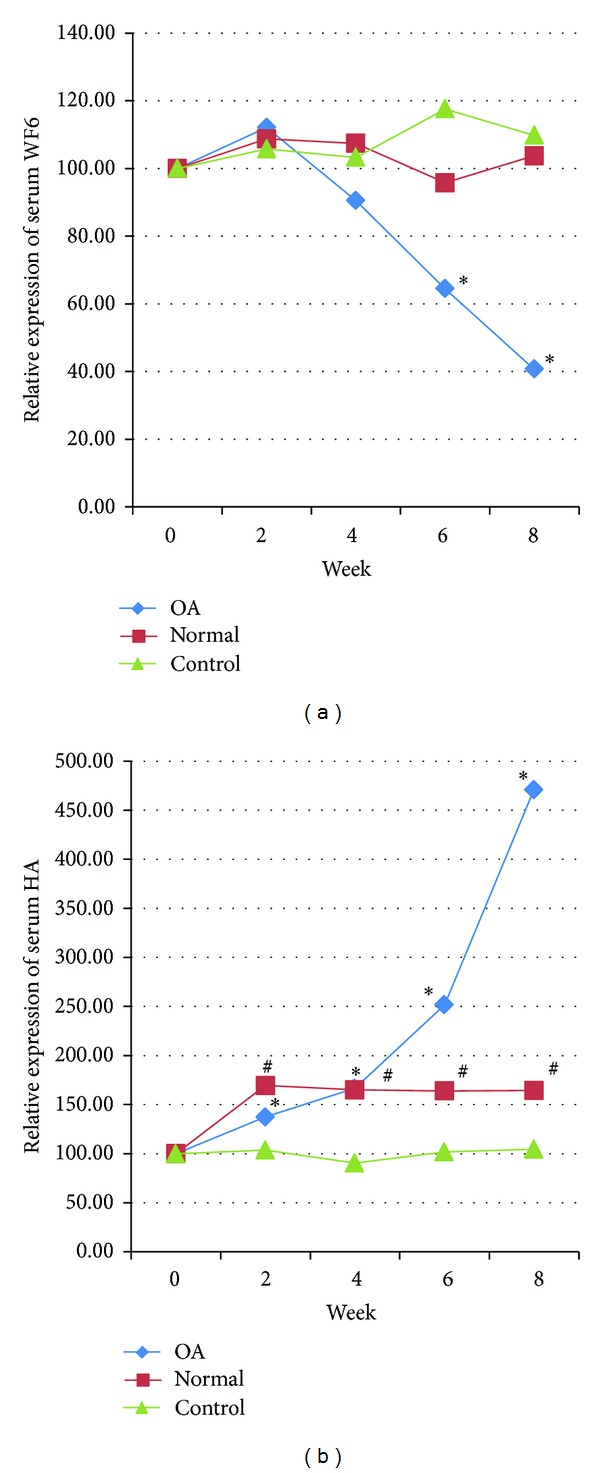
Mean of relative change (%) of serum chondroitin sulfate epitope WF6 (CS-WF6) and hyaluronan (HA). The symbols ∗ and # signify a significant difference within groups compared to week 0 (*P* < 0.05).

All 22 dogs in the OA-SW group had been diagnosed with OA of the hip joint and were classified as grade 1.95 ± 0.67 via a radiographic scoring system. Two biomarkers (CS-WF6 and HA) were also used to confirm OA by comparing OA and non-OA groups (Figure 1). The OA group showed significantly (*P* < 0.05) lower HA (31.25 ± 18.52) and higher CS-WF6 (83.91 ± 35.64) levels compared to the non-OA group (HA = 70.42 ± 27.97 and WF6 = 29.79 ± 24.66). The relative level of serum CS-WF6 in the OA-SW group was dramatically decreased beginning at week 4 (90.52 ± 31.02), but it was found to be significantly different (*P* < 0.05) compared with preexercise (100) level at weeks 6 (64.44 ± 23.16) and 8 (40.68 ± 19.71). On the other hand, the relative expression of serum CS-WF6 in the other two groups (H-SW and H-NSW) showed no significant change over the 8-week exercise period (Figure 2). The relative level of serum HA in the OA-SW group increased beginning at week 2 (137.50 ± 39.39) and then continued to rise steadily: at week 4, 166.60 ± 69.09; week 6, 257.75 ± 94.83; and at the end of week 8, 470.88 ± 286.96. Moreover, the levels of serum HA of the H-SW group were significantly (*P* < 0.05) higher than preexercise level: at week 2, 169.44 ± 102.44; week 4, 165.06 ± 55.87; week 6, 164.39 ± 75.28; and at the end of week 8, 164.39 ± 29.68 (Figure 3).

## 4. Discussion

The study design had several limitations. First, because this was a clinical study the animals could not be controlled by using the same breed, sex, and/or age. Moreover, not all dogs in the study had the same OA grade. However, we tried to maximize the number of animals (22) included in the OA with swimming group. Second, this study did not include an OA with non-swimming group. This is because all dogs in this study were pets with OA hip problems and had been brought to a small animal hospital by their concerned owners; for ethical reasons, it was felt that these animals should not be deprived of treatment to relieve pain. Third, since this study used an outdoor swimming pool, we were unable to do a long-term study (4 to 6 months or more) because the rainy season in the north of Thailand would overlap with the study period. Some animals swam for longer than 2 months, but only a small number which was insufficient for statistical analysis. So we established a 2-month cutoff period for studying the effects of the swimming program. (However, we have recently constructed an indoor swimming pool for future studies on the long-term effects of swimming on OA dogs.) Fourth, the total number of animals in this study was not large, particularly because many dogs (*n* = 22) withdrew from the study due to various problems: illness (10 dogs), moving out of the study area (5), death (2), and inability to swim frequently (12). Another possible limitation of the study is that we measured only the hip and no other joints.

Human studies have found that water temperature is another factor affecting physiology during aquatic exercise, for example, heart rate or blood pressure. Previous human studies showed higher heart rates during swimming in water with a temperature of 33°C versus 27°C or lower [25, 26]. (This is due to an increase in peripheral circulation from warmer water.) Although there are no existing reports on the effect of water temperature on canine physiology during swimming, our study was performed in water with a temperature between 30–35°C to avoid this effect of water temperature.

Another limitation in this study is that we did not have a force plate analysis instrument. Evaluation of clinical signs and range of motion of the hip joint were performed by two veterinarians via blind technique. Our trial found that the swimming program had a slow effect on clinical signs (lameness, joint mobility, weight bearing, and overall score), with improvement at week 8; only the pain on palpation score showed significant improvement earlier, at week 6. To evaluate the motion of the hip joint, passive ROM was measured every 2 weeks by two independent veterinarians. Swimming was found to improve the ROM of the hip joint not only in OA dogs but also in healthy dogs as well, with a significant improvement shown at week 8.

A previous study in humans also indicated that hydrotherapy can improve functional gains [27]. However, some research reports have had a different result. In 2003, [28] reported no significant effect of a 20-week aquatic training program on children with juvenile idiopathic arthritis. But that research had several limitations, for example, a limited number of patients, low intensity and frequency of exercise, and in-home assessment. Another advantage of swimming, from a recent study using a mouse model, is increased muscle mass, function and metabolic profile [29].

Based on the present results, it can be concluded that swimming 2 to 3 times per week for 8 weeks continuously can improve the ROM of the hip joint by about 5%, not only in OA dogs but also in normal dogs as well. A human study found that aquatic exercise for 6 weeks can improve the ROM of the hip joint by 10.9% [30]. A study comparing the therapeutic benefits of treadmill walking and swimming found that dogs that swam had significantly greater stifle ROM compared with dogs that exercised by walking on a treadmill [31].

Moreover, we used radiographic images to compare the pathology of OA joints between pre- and postexercise. Radiographic findings between weeks 0 and 8 in the OA-SW group showed no significant change. It showed that swimming can delay morphological change in particular joint space narrowing. However, radiographic images cannot provide as much information as an MRI or CT, but we did not have these facilities for animal use at our institute.

When the levels of serum biomarkers were compared between the OA and non-OA groups, a significantly lower level of serum HA and a higher level of serum CS-WF6 were found in OA dogs compared with non-OA dogs. This result was similar to our previous study on dogs with hip dysplasia [23], which showed that the serum levels of CS-WF6 increased, while HA levels decreased. Taken together with other reports [4, 21], this demonstrates the usefulness of these biomarkers to predict the progress of OA. An increase in the WF6 epitope may reflect a catabolic response, while a decrease may reflect a blockage of the catabolic pathways; this may be helpful for the diagnosis or prognosis of disease. The HA concentration in the joint fluid and serum of animals with diseased joints has been reported to be lower than normal because of a decrease in the synthesis mechanism via synoviocytes and chondrocytes [23, 32].

Hip joints are diarthrodial joints, which are freely moveable joints containing synovial fluid within a connective tissue joint capsule that allows for low-friction and low-wear articulation of the cartilaginous ends of long bones. The articular cartilage is a structure without blood vessels or nerve supply. Chondrocytes receive all nutrients and release waste products via the synovial fluid. Joint movement is very important for homeostasis in the joint environment because it helps articular cartilage absorb synovial fluid. In OA animals, joint movement is restricted because of pain, which in turn decreases the absorption of synovial fluid by articular cartilage. This will lead to decreased nutrients and an accumulation of waste products in cartilage. Shortly after exercise, an elevation of serum levels of cartilage oligomeric matrix protein (COMP) was found in patients with OA, suggesting an effect on cartilage metabolism [33]. A recent study found an exercise-induced increase in interleukin-10 levels in the (peri-)synovial fluid of patients with knee OA, to which anti-inflammatory and chondroprotective properties are ascribed [34].

A novel monoclonal antibody, WF6, which recognizes an epitope in native CS chains [24], was decreased after swimming. The finding of decreased levels of serum CS-WF6 after exercise reflects an alteration in the metabolism of the cartilage. In chronic OA, the level of CS-WF6 is higher than normal because the native CS chain in cartilage is degraded and released into the blood stream [23, 24]. The decrease of CS-WF6 in this study indicated that swimming could increase the anabolism and decrease the catabolism in OA joints. It is also possible that swimming could increase the blood supply to the joint, thus increasing the metabolism in cartilage and surrounding tissue. This is supported by the serum HA results in the present study; HA levels increased in both swimming groups, but to a greater extent in OA dogs than in normal dogs. HA is mainly produced by fibroblasts and other specialized connective tissue cells. Although HA is widely distributed throughout the body (umbilical cord, nasal cartilage, vitreum, cutis, and lymph nodes in the thorax), the highest concentration is found in synovial fluid and also in connective tissue such as the synovial membrane. Our results found that, after 8 weeks of a swimming regimen, the rate of HA synthesis was higher in OA dogs than in normal dogs. It is possible that swimming induced HA synthesis by synoviocytes and chondrocytes from increased blood supply to the joint. In human studies, blood flow during maximal exercise compared to resting conditions has been found to increase up to 20-fold on average, and in predominantly white muscles increases up to 80-fold have been reported [35].

One disadvantage of this study was that we could not measure biomarker levels in synovial fluid during swimming, which could provide useful information for further research, for example, on the levels of other serum biomarkers or gene expression.

In conclusion, the present study demonstrates that it is possible to evaluate the effects of exercise on articular cartilage. We discovered a significant change in serum biomarker levels in the group that performed swimming compared to the nonswimming group. This results show the beneficial effect that exercise has on patients with OA. Swimming appears to be a useful strategy for regaining movement and function in with OA joint.

## Figures and Tables

**Table 1:** Radiographic scoring system for assessing dogs with osteoarthritis.

Grade		Radiographic evaluation
0	Normal	Not affected
1	Mild	Doubtful narrowing of joint space and possible osteophytic lipping
2	Moderate	Definite osteophytes and possible narrowing of joint space
3	Severe	Moderate multiple osteophytes, definite narrowing of joints space, some sclerosis and possible deformity of bone contour
4	Very severe	Large osteophytes, marked narrowing of joint space, severe sclerosis and definite deformity of bone contour

**Table 2:** Clinical scoring system for assessing dogs with osteoarthritis.

Criterion	Grade	Clinical evaluation
Lameness	1	Walks normally
	2	Slightly lame when walking
	3	Moderately lame when walking
	4	Severely lame when walking
	5	Reluctant to rise and will not walk more than five paces

Joint mobility	1	Full range of motion
	2	Mild limitation (10–20%) in range of motion; no crepitus
	3	Mild limitation (10–20%) in range of motion; crepitus
	4	Moderate limitation (20–50%) in range of motion; ±crepitus
	5	Severe limitation (>50%) in range of motion; ±crepitus

Pain on palpation	1	None
	2	Mild signs; dog turns head in recognition
	3	Moderate signs; dog pulls limb away
	4	Severe signs; dog vocalizes or becomes aggressive
	5	Dog will not allow palpation

Weight bearing	1	Equal on all limbs standing and walking
	2	Normal standing; favors affected limb when walking
	3	Partial weight-bearing standing and walking
	4	Partial weight-bearing standing; non-weight-bearing walking
	5	Non-weight-bearing standing and walking

Overall score of clinical condition	1	Not affected
	2	Mildly affected
	3	Moderately affected
	4	Severely affected
	5	Very severely affected

**Table 3:** Comparison of clinical scores for the osteoarthritis-swimming (OA-SW) group before and during the experiment.

Parameter	Weeks				
	0	2	4	6	8
Lameness	3.00 ± 0.84^a^	2.95 ± 0.80^a^	2.95 ± 0.80^a^	2.86 ± 0.85^a^	2.48 ± 0.75^b^
Joint mobility	1.76 ± 0.83^a^	1.76 ± 0.83^a^	1.71 ± 0.78^a^	1.67 ± 0.73^a^	1.48 ± 0.60^b^
Pain on palpation	2.00 ± 0.55^a^	2.05 ± 0.59^a^	1.90 ± 0.62^a^	1.67 ± 0.58^b^	1.48 ± 0.51^b^
Weight bearing	2.05 ± 0.67^a^	2.00 ± 0.63^a^	1.95 ± 0.59^a^	1.90 ± 0.62^a^	1.48 ± 0.51^b^
Overall score	1.62 ± 0.59^a^	1.62 ± 0.59^a^	1.57 ± 0.60^a^	1.48 ± 0.60^a^	1.19 ± 0.40^b^

Data are expressed as mean ± SD.

A significant difference (*P* < 0.05) between the weeks at the same condition is displayed with superscript^(a,b)^.

**Table 4:** Comparison of the range of motion (ROM) of hip joint before and during the experiment.

Weeks	Group	Right hip joint		Left hip joint	
		Extension	Flexion	Extension	Flexion
0	OA-SW	128.24 ± 14.90^a^	41.14 ± 6.98^a^	128.52 ± 15.37^a^	40.81 ± 6.38^a^
	H-SW	137.00 ± 12.49^a^	41.27 ± 8.46^a^	137.33 ± 12.71^a^	41.40 ± 8.40^a^
	H-NSW	133.00 ± 7.49	38.77 ± 6.00	133.92 ± 7.68	39.46 ± 5.55

2	OA-SW	128.19 ± 15.24^a^	40.95 ± 7.04^a^	128.57 ± 15.13^a^	40.71 ± 6.47^a^
	H-SW	136.73 ± 12.74^a^	41.13 ± 8.33^a^	137.07 ± 12.07^a^	41.27 ± 8.51^a^
	H-NSW	133.08 ± 7.40	38.38 ± 5.92	133.77 ± 7.61	39.31 ± 5.69

4	OA-SW	128.62 ± 14.86^a^	40.86 ± 7.09^a^	129.05 ± 15.31^a^	40.52 ± 6.65^a^
	H-SW	137.33 ± 12.43^a,b^	41.00 ± 8.18^a^	137.60 ± 12.14^a^	40.93 ± 8.50^b^
	H-NSW	132.77 ± 7.5	38.69 ± 5.94	133.92 ± 7.53	39.54 ± 5.84

6	OA-SW	128.95 ± 15.05^a^	40.62 ± 6.57^a^	129.14 ± 15.63^a^	40.48 ± 6.71^a^
	H-SW	137.73 ± 12.69^b^	40.80 ± 8.42^a^	138.07 ± 12.33^a^	40.80 ± 8.41^b^
	H-NSW	132.93 ± 7.26	39.00 ± 6.18	134.00 ± 7.87	39.42 ± 5.64

8	OA-SW	130.48 ± 15.96^b^	40.00 ± 6.63^b^	130.43 ± 16.04^b^	39.38 ± 5.75^b^
	H-SW	139.53 ± 12.96^b^	40.33 ± 8.15^b^	140.02 ± 12.44^b^	40.27 ± 7.91^b^
	H-NSW	133.00 ± 7.57	38.62 ± 6.09	133.77 ± 7.61	39.85 ± 5.64

The data are expressed as mean ± SD.

A significant difference (*P* < 0.05) between the weeks (0, 2, 4, 6, and 8 weeks) at the same group is displayed with superscript^(a,b)^.
